# Evaluation of the vector competence of *Ixodes persulcatus* in the maintenance and transmission of Alongshan virus under laboratory conditions

**DOI:** 10.3389/fcimb.2026.1682683

**Published:** 2026-02-24

**Authors:** Zheng Gui, Yanan Wang, Liang Li, Yuanning Ren, Qiqi Guo, Ziyan Liu, Jingfeng Yu, Jinlin Zhou, Zedong Wang

**Affiliations:** 1 Department of Infectious Diseases, Center of Infectious Diseases and Pathogen Biology, State Key Laboratory of Zoonotic Diseases, the First Hospital of Jilin University, Changchun, Jilin, China; 2 Key Laboratory of Animal Parasitology of Ministry of Agriculture, Shanghai Veterinary Research Institute, Chinese Academy of Agricultural Sciences, Shanghai, China; 3 State Key Laboratory of Pathogen and Biosecurity, Changchun Veterinary Research Institute, Chinese Academy of Agricultural Sciences, Changchun, Jilin, China; 4 School of Basic Medicine, Inner Mongolia Medical University, Hohhot, Inner Mongolia, China; 5 International Center of Future Science, Jilin University, Changchun, Jilin, China

**Keywords:** Alongshan virus, *Ixodes persulcatus*, Jingmenvirus, tick-borne viruses, vector competence

## Abstract

**Background:**

Alongshan virus (ALSV) is an emerging tick-borne segmented flavivirus associated with human febrile illness, belonging to Jingmenvirus group in the family *Flaviviridae*. *Ixodes persulcatus* (*I. persulcatus*) has been considered as the competent vector of ALSV in the field studies; however, no experimental study has yet evaluated the vector competence of *I. persulcatus* in the maintenance and transmission of ALSV.

**Methods:**

*I. persulcatus* adult ticks were infected with ALSV via anal pore microinjection. salivary glands (SG), midgut (MG), and ovaries (OV) were collected and subjected to quantitative real-time polymerase chain reaction (RT-qPCR) for ALSV RNA detection. Ticks fed on Kunming (KM) or NOD-SCID IL2rg-/- (NTG) mice, and their eggs, molted progeny, and mice tissues were collected for ALSV RNA detection to evaluate vector competence in maintaining and transmitting ALSV.

**Results:**

Viral RNA levels in ALSV-microinjected ticks increased significantly, peaking at 7 d.p.i. Viral RNA in SG, MG, and OV tissues showed a significant upward trend over time, with higher copy numbers in MG than in SG and OV. Eggs, hatched larvae, engorged larvae, molted nymphs, engorged nymphs, and adults all exhibited high levels of viral RNA copies, ranging from approximately 10^6^ to 10^9^ copies/μL. Viral RNA was detected in the blood and tissues of mice bitten by ALSV-infected adult, larval, and nymphal ticks, with lower RNA copies in adult-bitten mice than in larvae- or nymph-bitten mice. Notably, viral RNA copy numbers were significantly higher in the blood and tissues of mice inoculated with ALSV and then bitten by ALSV-free immature ticks, compared to those receiving ALSV alone.

**Conclusion:**

The results demonstrate that *I. persulcatus* ticks are competent vectors for ALSV, capable of transmitting the virus both vertically and horizontally with high efficiency, with tick bite-induced modulation of viral levels in mice varying according to the developmental stage of the tick.

## 1 Introduction

Jingmenviruses (JMVs) represent a recently emerging viral group initially detected in *Rhipicephalus microplus* ticks sampled in China during 2010 (Qin et al., 2014). They are associated with both arthropods and vertebrates and are currently classified as provisional members of the genus *Orthoflavivirus* within the family *Flaviviridae*, primarily due to their distinct segmented genome organization and evolutionarily conserved protein functions (Ogola et al., 2025).

Most JMVs are associated with ticks and include viral species such as Jingmen tick virus (JMTV), Alongshan virus (ALSV), Yanggou tick virus, Takachi virus, and Baishan forest tick virus, with JMTV and ALSV having been confirmed to be associated with human febrile illnesses (Qin et al., 2014, Z. D. Wang et al., 2019a, Kholodilov et al., 2021; Kobayashi et al., 2021; Wang et al., 2023; Jia et al., 2019). The genome of tick-associated JMVs comprises four segments of single-stranded, positive-sense RNA (ss(+)RNA), where S1 and S3 encode nonstructural proteins (NSP1 and NSP2) that are homologous to the NS5 and NS3 proteins of classical orthoflaviviruses, while S2 and S4 encode structural proteins exhibiting no sequence homology to known viral proteins (Temmam et al., 2019; Valle et al., 2025).

ALSV was first identified in humans and *Ixodes persulcatu*s ticks in northeastern China in 2017 and has since been found in arthropods and mammals across Eurasia, including Russia, Finland, France, Germany, and Switzerland, indicating a broad host range and geographic spread, and a potential global public health threat (Gomer et al., 2024). The virus has been detected in various tick species worldwide, including *I. persulcatus*, *I. ricinus*, *Dermacentor nuttalli*, and *Haemaphysalis concinna* ticks (Gomer et al., 2024). Studies have confirmed that ALSV can replicate in *I. ricinus* and *D. reticulatus* ticks (Ebert et al., 2023). Additionally, effective viral transmission during blood feeding via the saliva of ALSV injected *I. ricinus* ticks was confirmed through *in vitro* feeding experiments.

In China, ALSV has primarily been detected in *I. persulcatus* ticks (Z. D. Wang et al., 2019a). Field studies have identified *I. persulcatus* as a competent vector for ALSV (Liu et al., 2022; Xu et al., 2024); however, no experimental study has yet evaluated the vector competence of *I. persulcatus* in maintaining and transmitting ALSV. In this study, ALSV was microinjected via the anal pore into *I. persulcatus* ticks to the vector competence of this tick species for ALSV transmission under laboratory conditions, providing new insights for the prevention and control of the virus.

## 2 Materials and methods

### 2.1 Ethics statement and biosafety

The animal studies were approved by the Animal Administration and Ethics Committees of the Changchun Veterinary Research Institute and the Shanghai Veterinary Research Institute, Chinese Academy of Agricultural Sciences (Approval numbers: AMMS-11-2020–026 and SHVRI-20230602-01). All procedures involving virus-infected or mock-infected ticks that had fed on mice were carried out in a designated containment area inside the animal biosafety level 2 (ABSL-2) facility. The studies were conducted in accordance with the local legislation and institutional requirements. Mice were handled humanely, anesthetized with an intravenous bolus of ethyl carbamate (100 mg/mL) at a dose of 1000 mg/kg based on body weight, and euthanized by carbon dioxide overdose. All procedures complied with the Animal Ethics Procedures and Guidelines of the People’s Republic of China.

### 2.2 Cells, virus, animals, and ticks

The *I. ricinus*-derived cell line (IRE/CTVM20) was obtained from the Tick Cell Biobank at the University of Liverpool and maintained at 30°C in L-15 (Leibovitz) medium supplemented with 10% tryptose phosphate broth, 20% fetal bovine serum (FBS; Sigma-Aldrich), 200mM L-glutamine, and 1% penicillin-streptomycin (Bell-Sakyi et al., 2007).

The ALSV NE-TH4 strain (NCBI accession numbers: ON408067–ON408070) was propagated in IRE/CTVM20 cells. Briefly, IRE/CTVM20 cells were seeded in flat-sided culture tubes (Nunc, Thermo Fisher Scientific) with 3 mL of complete medium and incubated at 30°C. Four days post-seeding, cells were infected by the addition of 100µL of ALSV culture supernatant and maintained at 30°C for an additional two weeks. The culture supernatants were collected and tested for ALSV RNA using RT-qPCR with primers detailed in Section 2.5. A focus-forming assay (FFU) was performed to quantify viral titers in the culture supernatants, as previously described (Rossi et al., 2012). Briefly, after infecting IRE/CTVM20 cell monolayers with 10-fold serial dilutions of virus in FBS-free medium, cells were incubated in medium containing 10% serum and supplemented with 0.8% carboxymethylcellulose for four days. Viral foci were visualized through a one-hour incubation with a 1:500 dilution of ALSV VP2-specific polyclonal antibody generated through rabbit immunization with recombinant VP2 protein as described in a previous study (Z. D. Wang et al., 2019b), followed by a one-hour incubation with an HRP-conjugated anti-rabbit secondary antibody (Abcam).

Eight-week-old KM mice were obtained from Liaoning Changsheng Biotechnology Co., Ltd. and used as a blood source for *I. persulcatus* ticks. NTG (NOD-SCID IL2rg−/−) mice, which are immunodeficient due to the absence of functional T, B, and NK cells, were acquired from Shanghai JieSiJie Laboratory Animal Co., Ltd. and utilized to establish the ALSV infection model. Following one week of acclimatization in ventilated cages under controlled temperature and humidity conditions, all mice were used in subsequent experiments.

The laboratory strain of *I. persulcatus* was maintained in a climate chamber under controlled conditions, with a humidity level of 95% and a temperature of 25 °C, and exposed to a 12-hour light-dark cycle. The ticks were fed on KM mice to sustain population maintenance and reproduction. Prior to experimental use, all ticks underwent next-generation sequencing to confirm the absence of ALSV and other potential microorganisms, including endogenous viruses.

### 2.3 Anal pore microinjection and tick dissections

Adult female *I. persulcatus* ticks were randomly allocated from an ALSV-free colony into infected and control groups. The infected group was microinjected with 500 nL of a solution containing approximately 100 FFU of ALSV, whereas the control group received an equivalent volume of IRE/CTVM20 cell supernatant. Microinjections were administered via the anal pore using the IM 300 Microinjector (NARISHIGE). At 1, 3,5,7, and 9 days post-infection (d.p.i.), five adult ticks were randomly collected from both the ALSV-injected and mock-injected groups for viral detection as intact whole individuals. Additionally, three biological replicates, each comprising five ticks, were randomly selected from both groups; these were dissected to harvest and pool salivary glands (SG), midgut (MG), and ovaries (OV) for ALSV RNA detection.

### 2.4 RNA extraction and cDNA synthesis

Ticks or mice tissues were homogenized in 300 µL of TRIzol reagent with sterile metal beads using an ultrasonic crusher (MB-LD48S) at 55 Hz for 5 minutes. After adding an additional 700 µL of TRIzol reagent, the homogenate was incubated at room temperature for 10 minutes. Furthermore, RNA was extracted using an automated nucleic acid extraction kit (Shanghai manman biotechnology Co., Ltd) and reverse-transcribed into complementary DNA (cDNA) using HiScript III RT Surper Mix for qPCR (Vazyme, China), following the manufacturer’s instructions.

### 2.5 Quantitative real-time polymerase chain reaction

Viral cDNA in tick or mice samples was detected using RT-qPCR, with primers forward: (5’- GCTTGTGGTCATCATTATG-3’), reverse: (5’-CTCTGCCACATACTGATG-3’), and a probe (FAM-CTCTCGTCAGCCATACCACCA-BHQ-1) specific to ALSV S2 segment, following the manufacturer’s instructions of the Premix Ex Taq (Probe qPCR) Kit (TaKaRa, Japan). The thermal cycling protocol included an initial step of denaturation at 95°C for 30 seconds, then proceeded with 50 cycles consisting of denaturation at 95°C for 5 seconds and annealing/extension at 60°C for 30 seconds. The viral RNA copy numbers were determined using a formula derived from a standard curve generated from tenfold serially diluted recombinant plasmids: (copies)/μL = 10^ [(Ct value − 39.01)/−2.895] (**Supplementary Figure S1**).

### 2.6 Vertical and horizontal transmission of ALSV

To evaluate the transovarial transmission competence of ALSV in *I. persulcatus*, three ALSV-microinjected ticks were allowed to feed on 8-week-old male KM mice at 7 d.p.i. using the model described in the previous study (Wang et al., 2024). After feeding on mice for approximately seven days, the engorged ticks were individually housed under controlled conditions of 25°C and 90% humidity to promote oviposition and ensure successful hatching. At 30 days post-oviposition, three pools of 50 eggs each were collected from each egg mass for ALSV-RNA detection. The remaining eggs were permitted to hatch into larvae, and three additional pools, each containing 20 tick larvae, were collected for ALSV-RNA detection.

To assess the transstadial transmission competence of ALSV in *I. persulcatus*, the remaining tick larvae were allowed to feed on KM mice, with approximately 200 tick larvae per mouse. Three pools of 10 engorged larvae each were collected for ALSV-RNA detection. The remaining engorged larvae were maintained until molting into nymphs, and 30 nymphs, grouped into three pools, were subsequently collected for ALSV-RNA detection. The remaining nymphs were then allowed to feed on mice, with approximately 50 nymphs per mouse; three engorged nymphs were individually collected and tested for ALSV RNA. The remaining engorged nymphs were maintained under conditions permitting molting into second-generation adult ticks over a period of two months, and three adult ticks were collected and individually tested for ALSV RNA.

To investigate the horizontal transmission of ALSV from ticks to mice, KM mice that had been fed upon by adult ticks, larvae, and nymphs confirmed to be ALSV-RNA positive were euthanized, and their hearts, brains, livers, kidneys, lungs, spleens, testicles, and blood were collected for ALSV RNA detection (**Figure 2A**).

### 2.7 Acquisition of ALSV by ticks feeding on viremic mice

To establish ALSV-viremic mice, eight-week-old NTG female mice were intraperitoneal inoculated with 10^6^ FFU of ALSV. At 3 and 7 d.p.i., three mice were euthanized at each time point to collect hearts, brains, livers, kidneys, lungs, spleens, intestines, and testes for confirmation of the copy number of ALSV infection. To investigate the acquisition competence of ALSV from mice, ALSV-free *I. persulcatus* larvae (50 per mouse) and nymph ticks (15 per mouse) were allowed to feed on ALSV-infected or mock-infected female mice (three mice per group) at 3 d.p.i. The engorged larval (5 engorged larvae per group) and nymphal (an engorged nymph per group) ticks were harvested and stored at -80 °C until ALSV detection.

### 2.8 Statistical analysis

Statistical analysis was performed using GraphPad Prism 9.0. For normally distributed datasets, significance was assessed by one-way ANOVA or two-way ANOVA with Tukey’s test. Significance is indicated by asterisks: *p < 0.05; **p < 0.01; ***p <0.001; ****p < 0.0001.

## 3 Results

### 3.1 ALSV infection in microinjected ticks and their tissues

**Figure 1:**
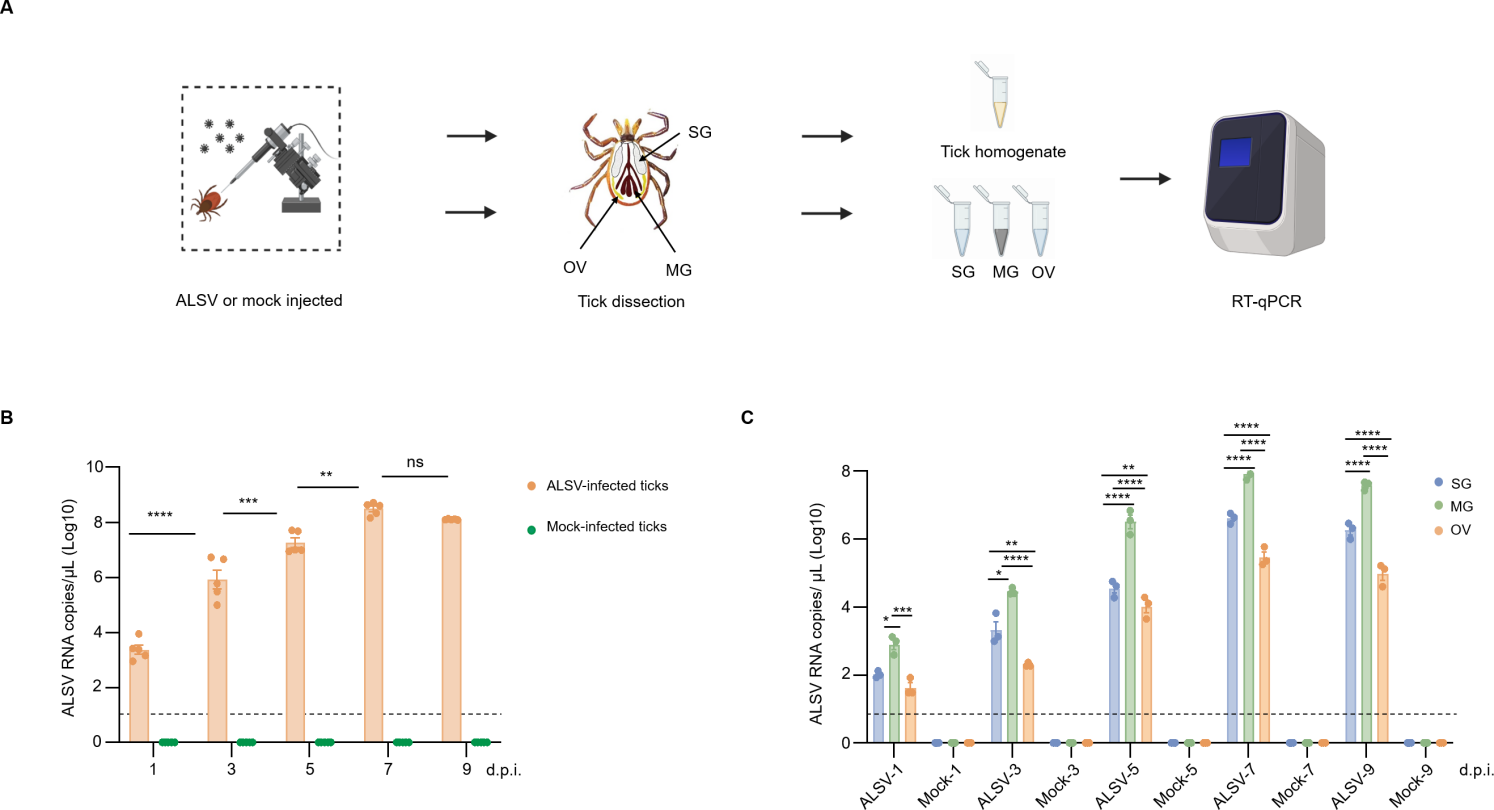
Acquisition and maintenance of ALSV in ticks via anal pore microinjection. **(A)** Schematic of ALSV infection in anal-pore microinjected ticks. **(B)** ALSV copy numbers in microinjected ticks (10^3^–10^9^ copies/mL). Each data point representative of an individual (n=50), mock-injected groups were negative for ALSV RNA. **(C)** Comparison of ALSV copy numbers in tick salivary glands (SG) (10^1^–10^7^ copies/mL), midgut (MG) (10^2^–10^8^ copies/mL), and ovary (OV) organs (10^1^–10^6^ copies/mL). Each data point representative of a pool of 5 individuals (n=150), three replicates presented on one bar. The limit of detection of ALSV is 10 copies/mL. * indicates p < 0.05, ** indicates p < 0.01, *** indicates p < 0.001, **** indicates p < 0.0001. ALSV: ALSVinfected ticks, Mock: Mock-infected ticks.

As shown in the flowchart (**Figure 1A**), both microinjected intact ticks and their corresponding tissues (SG, MG, and OV) were subjected to ALSV RNA detection. For the ALSV-injected ticks, viral RNA levels increased significantly, peaking at 7 d.p.i. (**Figure 1B**). Viral RNA in SG, MG, and OV tissues showed a significant upward trend over time, indicating that the virus can replicate in all three tissues (**Figure 1C**). Moreover, the copy numbers of ALSV in the MG at 1, 3, 5, 7 and 9 d.p.i. were significantly higher than those in the SG and OV. No ALSV RNA was detected from the control group.

### 3.2 Vertical transmission of ALSV

**Figure 2:**
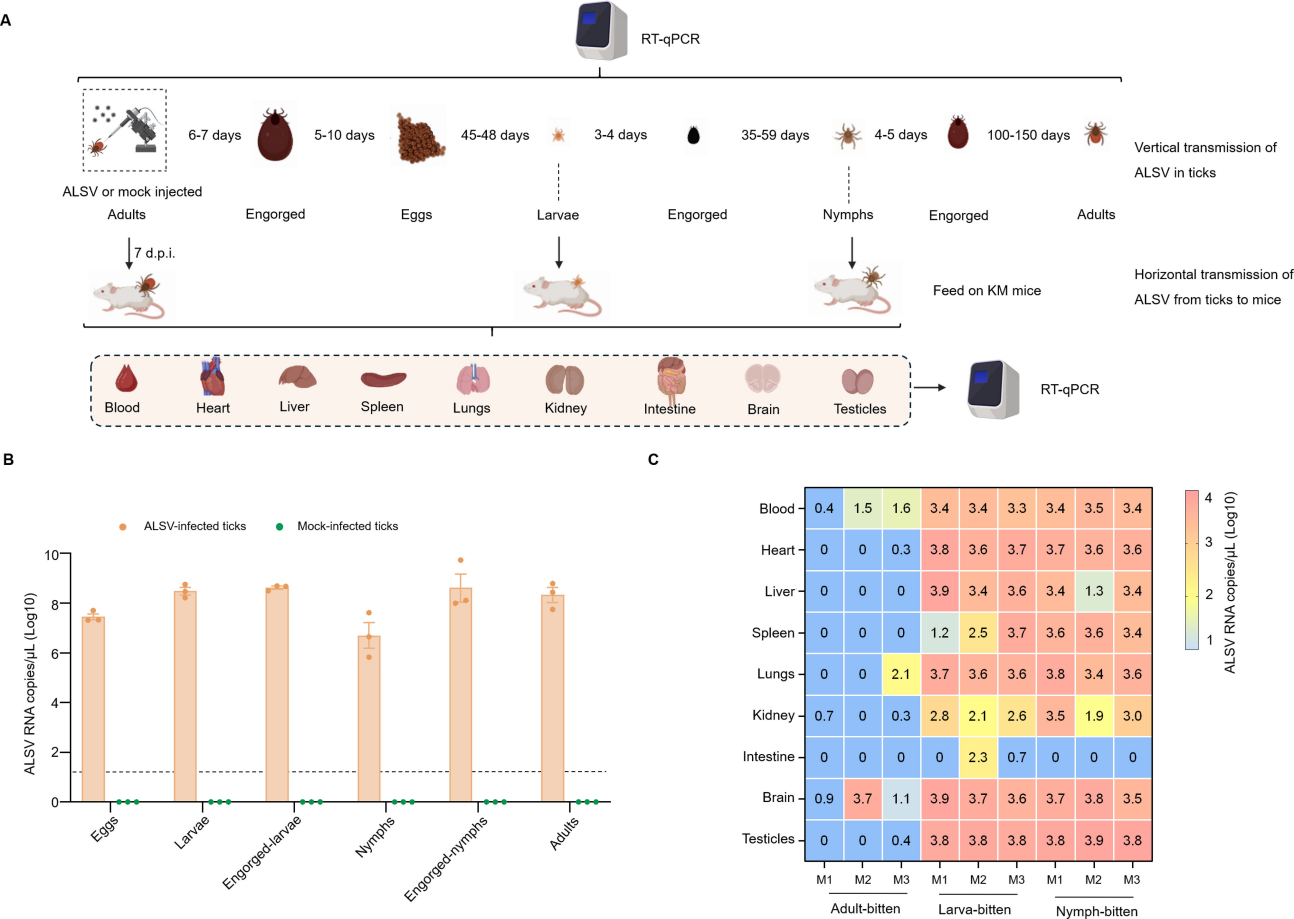
Vertical transmission of ALSV in ticks and horizontal transmission of ALSV from ticks to mice. **(A)** Schematic of vertical transmission of ALSV in ticks and horizontal transmission of ALSV from ticks to mice. **(B)** Transovarial and transstadial transmission analysis of ALSV copy numbers in offspring eggs (50 eggs each pool), larvae (20 larvae each pool), engorged larvae (10 engorged larvae each pool), nymphs (10 nymphs each pool), engorged nymph (one nymph per group) and adults (one adult per group). The limit of detection of ALSV is 10 copies/mL (The value ≤ 1 (Log10) showed in the heatmap). **(C)** Horizontal transmission of ALSV from ticks to mice. ALSV copy numbers in mice bitten by adults, larvae and nymphs were from 10^0^–10^4^ copies/mL. M1: Mice 1; M2: Mice 2; M3: Mice 3.

As shown in the flowchart (**Figure 2A**), transovarial and transstadial transmission experiments were conducted to assess the vertical transmission competence of ALSV in *I. persulcatus*. Both the eggs and the hatched larvae exhibited high RNA copy numbers, ranging from 10^7^ to 10^8^ RNA copies/μL, indicating that ticks are capable of transmitting ALSV transovarially. Although no significant difference in RNA copy numbers was detected between eggs and larvae, the slightly higher levels in larvae suggest possible ALSV replication in larval ticks. ALSV RNA were also detected in engorged-larvae, nymphs, engorged-nymphs, and adult ticks with high RNA copies various from 10^6^ to 10^9^ copies/μL. It is worth noting that the virus titers in both nymph and adult ticks that molted from engorged-larvae or nymphs were lower than those prior to molting. Although the difference was not statistically significant, this suggests that the virus transstadial transmission in *I. persulcatus* ticks may face certain challenges. No ALSV RNA was detected from the control group (**Figure 2B**).

### 3.3 Horizontal transmission of ALSV from ticks to mice

Adult, larval, and nymphal ticks infected with ALSV were fed on female KM mice and subsequently removed after engorgement. The mice that were bitten by ticks were sacrificed to collect blood and organ tissues for the detection of ALSV. Viral RNA can be detected in the blood and tissues of mice bitten by ticks at various life stages (**Figure 2A**). It is noteworthy that the RNA copy numbers in the blood and tissues of mice bitten by adult ticks were significantly lower than those in mice bitten by larvae or nymphs. In mice bitten by adult ticks, viral RNA was detectable in the blood and brain of all three animals. However, viral RNA copies in other tissues were low and rarely detectable. In mice bitten by larvae or nymphs, viral RNA at higher titers (10^3^ to 10^4^ copies/μL) was detectable in the blood and nearly all tissues, with the exception of the intestine (**Figure 2C**).

### 3.4 Acquisition of ALSV by ticks feeding on viremic mice

**Figure 3:**
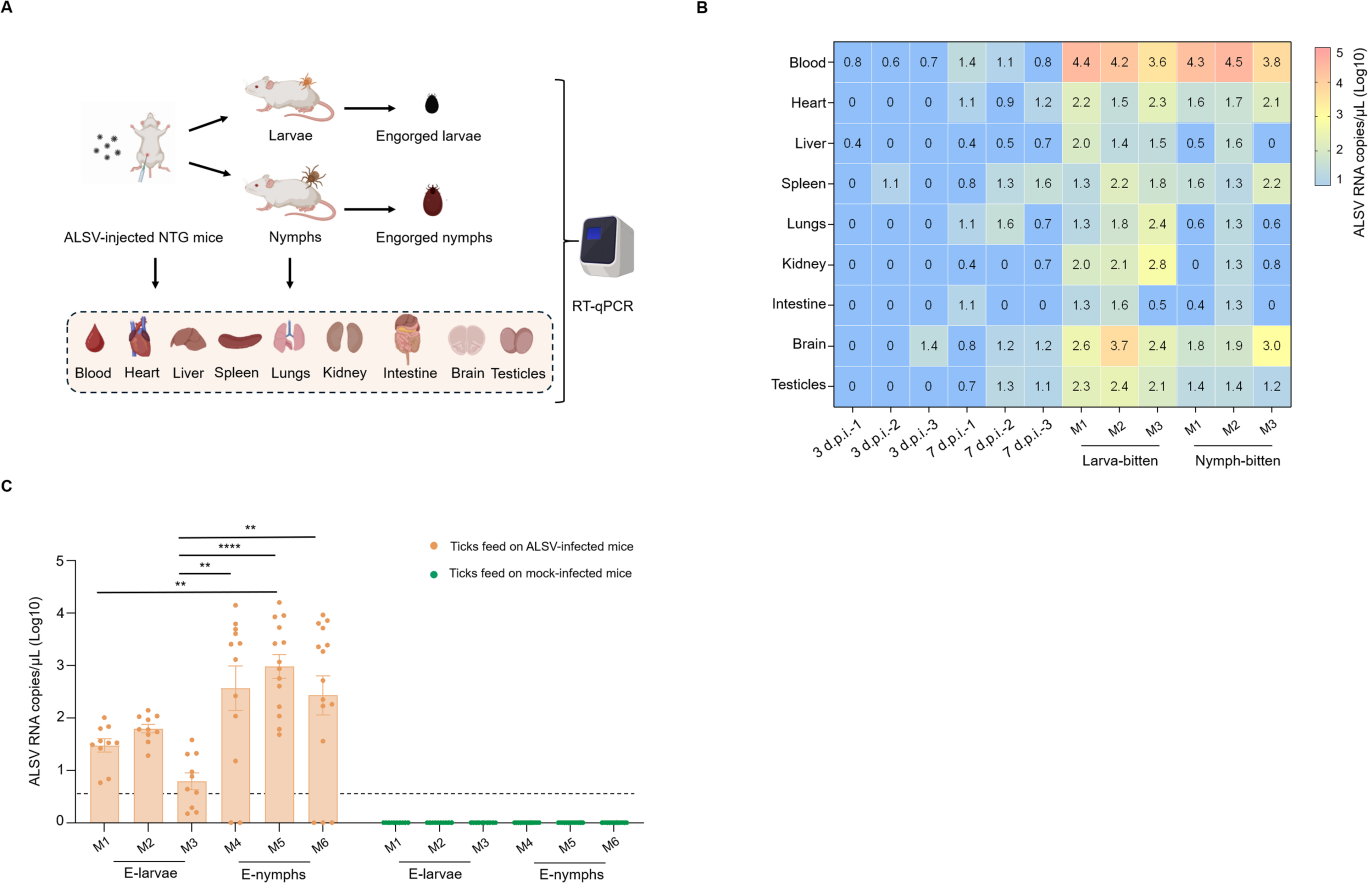
ALSV acquisition by immature ticks feeding on viremic mice. **(A)** Schematic of ALSV acquisition by immature feeding on viremic mice. **(B)** ALSV copy numbers in blood and tissues of NTG mice intraperitoneally injected either solely or ticks subsequently bitten by immature ticks. **(C)** ALSV copy numbers in engorged larvae (n=150, 10^1–^10^2^ copies/μL) and nymphs (n=41, 10^3–^10^5^ copies/μL). The limit of detection of ALSV is 10 copies/μL (The value ≤ 1 (Log10) showed in the heatmap). Ticks feed on mock-infected mice were negative for ALSV RNA. ** indicates p < 0.01, *** indicates p < 0.001, **** indicates p < 0.0001. E-larvae: Engorged-larvae, E-nymphs: Engorged-nymphs. M1: Mice 1; M2: Mice 2; M3: Mice 3. M4: Mice 4; M5: Mice 5; M6: Mice 6.

After feeding on ALSV-inoculated female NTG mice, engorged larvae, nymphs, and mouse blood/tissues were collected for ALSV RNA detection (**Figure 3A**). In mice intraperitoneally inoculated with ALSV but not bitten by larval or nymphal ticks, low levels of viral RNA were detected in blood at both 3 and 7 d.p.i. At 7 d.p.i., a broader tissue distribution with slightly higher RNA copy numbers was observed. Considering the 3- to 5-day blood-sucking cycle of immature ticks (larvae and nymphs), tick feeding was allowed to occur at 3 d.p.i. Notably, viral RNA copy numbers were significantly higher in the blood, heart, liver, spleen, lung, kidney, intestine, and testicular tissues of mice inoculated with ALSV and then bitten by ALSV-free immature ticks, compared to those receiving ALSV alone at 3 d.p.i. A similar increase was observed in mice that received viral inoculation followed by bites from ALSV-free larvae, compared to those receiving ALSV alone at 7 d.p.i. No significant differences were observed between groups bitten by ALSV-free larvae versus nymphs (**Figure 3B**). Viral RNA was also detected in the collected engorged larvae and nymphs, with RNA copy numbers in engorged nymphs significantly higher than those in engorged larvae (**Figure 3C**). As expected, none of the engorged larvae or nymphs attached to mock-infected mice tested positive for ALSV.

## 4 Discussion

In this study, we successfully demonstrate that *I. persulcatus* ticks have the vector competence for both vertical and horizontal transmission of ALSV, which is consistent with our previous field investigation results, indicating that *I. persulcatus* is the predominant tick species carrying ALSV (Z. D. Wang et al., 2019a, Xu et al., 2024; Liu et al., 2022).

Using microinjection method, this study confirmed that adult *I. persulcatus* can acquire and maintain ALSV, vertical transmission of the virus from female ticks to their progeny, and horizontal transmission of the virus between ticks and mice. Currently, the primary methods for artificially infecting ticks with viruses include microinjection and immersion in viral suspension (Raney et al., 2022; Jia et al., 2019). The immersion method is straightforward and does not require specialized technical expertise or equipment. However, it is challenging to accurately assess the initial viral infection titer, and it is also difficult to ascertain whether the tick becomes infected via ingestion or through other routes such as the spiracle. Although the microinjection method via the anal pore is technically more demanding, it allows for precise quantification of viral infection and confirms that the alimentary canal is the primary site of initial infection (Raney et al., 2022). Anal pore microinjection better simulates natural infection, as the midgut is the first organ to acquire the virus during natural feeding (Maqbool et al., 2022).

Studies have reported that ALSV can effectively replicate in *I. ricinus* following microinjection with high level ALSV RNA (10^6^ copies/tick) instead of low level (10^3^ copies/tick) (Ebert et al., 2023). Furthermore, ALSV has also been confirmed to be excreted via *I. ricinus* tick saliva during the blood meal. Our study reveals that even following microinjection with a low viral titer, ALSV can efficiently replicate in *I. persulcatus*. Viral RNA levels exhibited a significant upward trend in ALSV-microinjected ticks and their salivary glands, midguts, and ovaries, indicating that ALSV can efficiently replicate within *I. persulcatus* (**Figure 1**). Moreover, our results demonstrated that the viral titer in the midgut has consistently remained higher than that in the ovaries and salivary glands since 1 d.p.i., suggesting that ALSV may be transmitted from the midgut to the salivary glands and ovaries in ticks.

Although anal pore microinjection allows for a more accurate simulation of natural infection, the process itself diverges from the natural route of transmission. In nature, ticks acquire the virus by feeding on the blood of viremic hosts, whereas microinjection involves direct introduction of the virus into the tick. Artificially infected ticks may encounter challenges such as failure to establish a stable infection. Here, we first employed artificial infection to determine the optimal time point for high ALSV RNA levels, and subsequently used viremic mice at the best time point to mimic the natural transmission cycle, thereby minimizing the impact of artificially simulated infections on subsequent experiments to the greatest extent possible.

The results from this study demonstrate that *I. persulcatus* is competent in transmitting ALSV via vertical transmission, including both transovarial and transstadial routes. The continuously increasing viral titers at high levels in ovaries, eggs, and hatched larvae confirm that ALSV can efficiently overcome the barrier of transovarial transmission (**Figure 1C**, **Figure 2B**). The decreased titer of ALSV in molted nymphs and adult ticks compared to that before molting indicates that it is challenging for ALSV to overcome the barrier of transstadial transmission, with some viruses being eliminated during this process (**Figure 2C**). The same phenomenon was observed in tick-borne encephalitis virus-infected *I. ricinus* immature ticks under laboratory conditions, the loss of infection during molting could have a significant impact on estimates of the basic reproductive number for tick-borne viruses (Slovak et al., 2014). This phenomenon may be one of the factors contributing to the low incidence of tick-borne virus infections in field-collected ticks.

To date, no successful mouse infection model for ALSV has been established. The primary reason is that ALSV fails to establish a high-titer, persistent infection in both wild-type and immunodeficient mice, and infected animals do not develop clinical symptoms or mortality. In this study, the NTG immunodeficient mouse infection model was suboptimal, as the virus maintained only low-level viremia (**Figure 3B**). However, this was sufficient to enable larval and nymphal ticks to successfully acquire ALSV from the blood meal (**Figure 3C**). Notably, viral RNA copy numbers were significantly higher in mice that received intraperitoneal ALSV inoculation and were then bitten by ALSV-free immature ticks, compared to those inoculated with ALSV alone (**Figure 3B**). This finding suggests that tick-biting may enhance ALSV infection and replication in mice. We speculate that bioactive components in the saliva of *I. persulcatus* ticks might contribute positively to this effect. For instance, numerous tick salivary proteins, including Serpins, Hyalomins, Japanin, Salp15, Salp20, Lipocalins, Metalloproteases, and Cystatins, have been shown to modulate the host immune response through diverse mechanisms (Gao et al., 2024). More studies should focus on how *I. persulcatus* salivary proteins facilitate ALSV transmission in hosts.

*I. persulcatus* poses a significant public health threat to the Eurasian continent, particularly in Russia, China, Japan, and several Baltic Sea countries. This tick species exhibits a broad host range (at least 46 host species) and can carry a wide variety of tick-borne pathogens (at least 51 agents) (Wang et al., 2023). Efficient surveillance of the vector competence for transmitting various *I. persulcatus*-borne pathogens will facilitate a deeper understanding of pathogen transmission mechanisms and enhance the formulation and implementation of prevention and control strategies.

In conclusion, our results suggest that *I. persulcatus* ticks are competent vectors of ALSV, capable of transmitting the virus both vertically and horizontally under laboratory conditions. We found that there is an influence of tick bites on ALSV levels in mice which is dependent on the tick developmental stage. Further research on the biology and ecology of *I. persulcatus*, as well as pathogen dynamics within these ticks in natural environments, will be essential for understanding the public health implications of ALSV.

## Data Availability

The raw data supporting the conclusions of this article will be made available by the authors, without undue reservation.
