# Supplementary material for: Evaluation of the vector competence of Ixodes persulcatus in the maintenance and transmission of Alongshan virus under laboratory conditions
Source: Front Cell Infect Microbiol. 2026 Feb 24;16:1682683. doi: 10.3389/fcimb.2026.1682683 (PMC12971710; doi:10.3389/fcimb.2026.1682683)
Supplement: Supplementary file 1 [file Table1.docx]

**Supplementary Material**


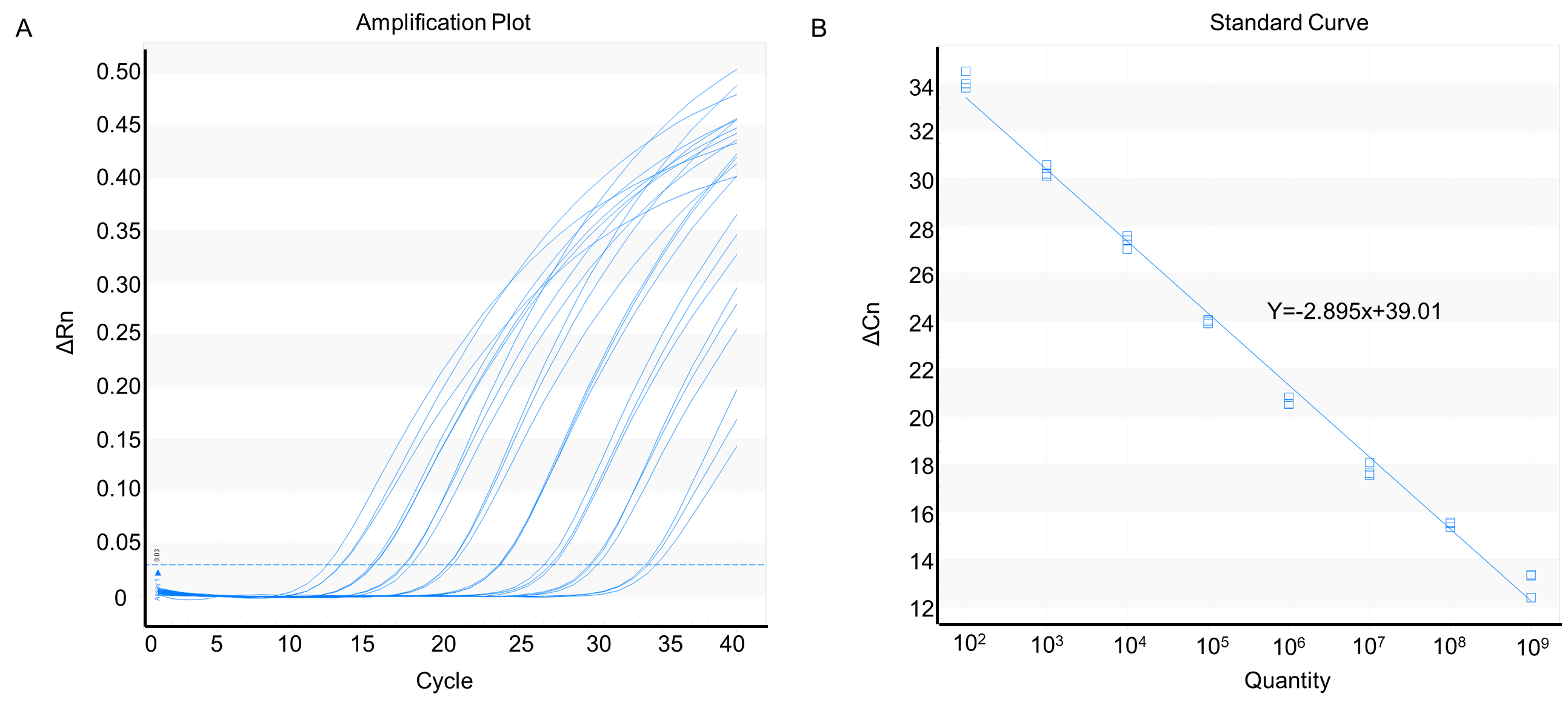


**Figure S1. Amplification plot and standard curve of the ALSV-specific Taqman RT-qPCR assay.**
